# Biochips for Direct Detection and Identification of Bacteria in Blood Culture-Like Conditions

**DOI:** 10.1038/s41598-017-10072-z

**Published:** 2017-08-25

**Authors:** V. Templier, T. Livache, S. Boisset, M. Maurin, S. Slimani, R. Mathey, Y. Roupioz

**Affiliations:** 1 Univ. Grenoble Alpes, CNRS, CEA, INAC, SYMMES, F-38000 Grenoble, France; 2 Laboratoire de Bactériologie, Centre Hospitalier Universitaire Grenoble Alpes, Univ. Grenoble Alpes, F-38000 Grenoble, France; 3 Equipe Therex, TIMC-IMAG, CNRS UMR 5525, Univ. Grenoble Alpes, F-38000 Grenoble, France; 4 Present Address: CREAB, SyMMES – UMR 5819, INAC CEA-GRENOBLE, 38054 Grenoble Cedex, France

## Abstract

Bloodstream bacterial infections are life-threatening conditions necessitating prompt medical care. Rapid pathogen identification is essential for early setting of the best anti-infectious therapy. However, the bacterial load in blood samples from patients with bacteremia is too low and under the limit of detection of most methods for direct identification of bacteria. Therefore, a preliminary step enabling the bacterial multiplication is required. To do so, blood cultures still remain the gold standard before bacteremia diagnosis. Bacterial identification is then usually obtained within 24 to 48 hours -at least- after blood sampling. In the present work, the fast and direct identification of bacteria present in blood cultures is completed in less than 12 hours, during bacterial growth, using an antibody microarray coupled to a Surface Plasmon Resonance imager (SPRi). Less than one bacterium (*Salmonella enterica* serovar Enteritidis) per milliliter of blood sample is successfully detected and identified in blood volumes similar to blood tests collected in clinics (*i.e*. several milliliters). This proof of concept demonstrates the workability of our method for human samples, despite the highly complex intrinsic nature of unprocessed blood. Our label-free method then opens new perspectives for direct and faster bacterial identification in a larger range of clinical samples.

## Introduction

Bacteremia is defined as the presence of viable bacteria in bloodstream, a normally sterile site. It might be a very transient event as, in most cases, bacteria are quickly cleared by the human immune system without occurrence of any symptoms. In contrast, persistent and life-threatening bacteremia may occur in patients with particular risk factors such as previous infections, presence of intravascular catheters or weakened immune system^1^. Young children, elderly persons and people with poor health conditions are more likely to develop persistent bacteremia^1, 2^. Bacteremia more frequently occur in hospital settings, especially in intensive care units, but may also affect sub-populations^3^. Persistent bacteremia are most often associated with sepsis, a physiological state that worsens the infection prognosis as death rates ranging from 20% to 50% as well as severe long-term consequences for survivors are frequently encountered^4^. Complete mechanisms of sepsis are not totally understood so far^5^ although a well-known triggering event is the production of cytokines by the immune system stimulated by conserved bacterial motifs^6^. Sepsis containment requires bacterial multiplication control as early as possible. In most cases, a wide-spectrum antibiotic therapy is administrated to patients with a suspected sepsis before full microbiological data are available, then narrow-spectrum antibiotics might be delivered according to the involved pathogen^7^.

Bacterial loads found in blood drawn from patients with bacteremia rarely exceed 10 CFU.mL^−1^ and are usually below 1 CFU.mL^−1^ ^8, 9^. Whatever the detection methods, including molecular methods, none of them is able to detect such a low number of bacteria on raw samples^10^. Then, after sampling, blood is diluted and incubated for one to several days in blood culture bottles containing enriched liquid culture media enabling pathogens growth and multiplication^8, 9^, even in the presence of residual antibiotics^11, 12^. This dilution in culture media is mandatory as it enables both antibiotics neutralization (usually on active carbon) and optimal growth conditions for contaminating bacteria. In this way, pathogen concentrations obtained after culture usually exceed the limit of detection of the following analysis protocol. Moreover, sensitivity of most methods used for bacterial identification is strongly limited by the blood sample volume^8^. For these reasons, 20 mL to 60 mL of blood are usually drawn to get enough bacteria in the sample to be cultured. Besides the concentration issue, the natural intrinsic molecular and cellular complexity of blood also makes bacterial detection difficult in such unprocessed body fluid.

Usually, when a blood culture bottle turns positive after enrichment, a Gram stain is performed and blood culture supernatants are spread on agar plates and cultured to obtain individualized bacterial colonies. Bacterial identification may then be performed at best after a 24 hours-incubation^13, 14^ but usually at least 48 hours are required for antibiotic susceptibility testing using automated systems such as Vitek® 2 (bioMérieux) or BD Phoenix™ (Becton Dickinson). The whole procedure then takes at least 48 hours - and up to several days depending on the pathogen- to get both the bacteria identification and antibiotic susceptibilities. Decreasing the diagnostic delay is then of paramount importance to enable earlier delivery of the most convenient anti-infectious therapy and eventually significantly improve patient prognosis.

To that end, an increasing number of emerging methods, appearing as alternatives to standard protocols, are currently under development. Based on the high bacterial concentrations obtained after enrichment, as positive blood cultures usually contain at least 10^7^–108 CFU.mL^−1^ ^15, 16^, bacteria identification may be led on culture supernatants using MALDI TOF mass spectrometry^17, 18^ or other automated molecular methods^19^. Such approaches are promising but still need to be further improved to better detect some bacterial strains. Nucleic-acid-based methods may also be performed on positive blood culture supernatants after enrichment, with a processing time generally shorter than 2 hours and good sensitivity and specificity values. However, these label-based molecular techniques remain labor-intensive and expensive. Alternative techniques relying on molecular methods such as fluorescence *in situ* hybridization (FISH), DNA microarrays hybridization or real-time PCR are also currently evaluated^16, 20^. However, in most cases, if any, a pre-enrichment step is mandatory and assay completion remains labor extensive. Some PCR-based methods have been directly applied to unprocessed blood samples, but showed poor sensitivity^10, 16^. As an example, the real-time PCR test SeptiFast® (Roche Diagnostics, Meylan, France) displays a sensitivity of 50% compared to regular methods carried after blood culture^10^. Finally, the clinical significance of bacterial DNA detection is debatable as false-positive results may be due to dead microorganisms or DNA traces released after pathogen neutralization by the immune system or antibiotic therapy.

Surface Plasmon Resonance imaging (SPRi) is an optical detection technique enabling the real-time and label-free monitoring of molecular interactions occurring on metallic layers. Recently, SPRi has been coupled to protein microarrays to detect food-related bacterial pathogens in small (about 1 mL) sample volumes. The successful detection of live bacteria was possible by choosing antibodies specific to antigens expressed by the bacteria and located on the cell surface^21, 22^. Although this approach showed promising results for the detection of low levels of contaminating bacteria present in optimized liquid culture media or foodstuff, its applicability has never been proved so far, neither for bacterial detection in large volume of blood nor prior to bacterial multiplication during blood culture^23^. The results described in the present manuscript confirm the efficiency of such label-free optical method for the monitoring of multiplying bacteria in diluted human blood processed on protein microarrays. As a proof of concept of the workability of this one-step approach in biomedical conditions, we carried the direct detection and identification of clinically relevant levels of *Salmonella enterica* serovar Enteritidis contaminating human blood and serum, during bacterial growth, using an antibody array.

## Results

**Figure 1:**
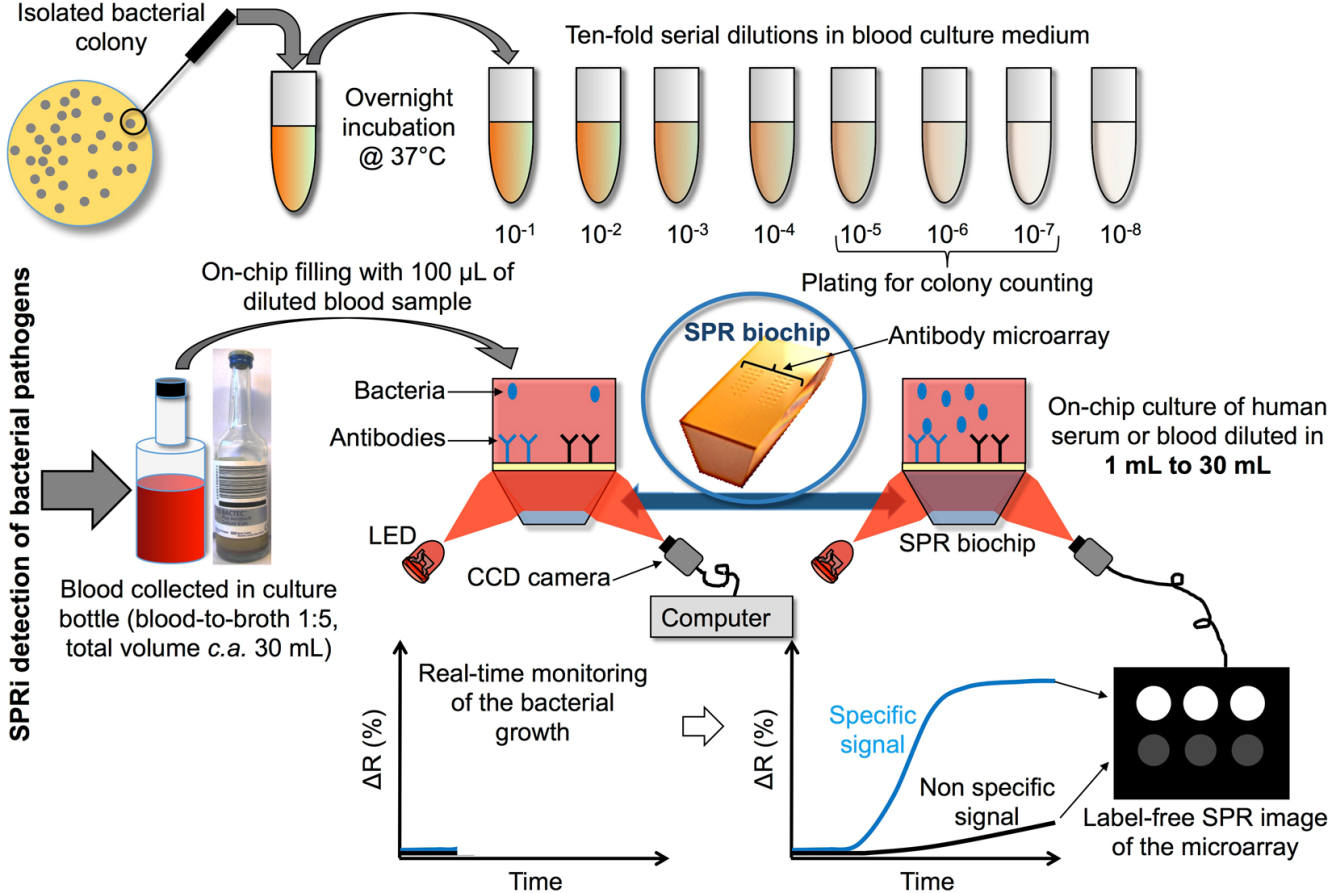
Label-free detection of bacteria in blood using protein microarrays and SPRi detection. Bacteria from an isolated bacterial colony were grown overnight in blood culture medium. Then, the culture was ten-fold serially diluted in the same medium and a diluted aliquot was finally mixed with blood samples so that the blood-to-broth ratio was kept constant (1:5) similarly to conditions routinely used in clinical settings. Several dozens of antibodies can be arrayed on 1 cm^2^. Live bacteria are captured on microarrayed specific antibodies (spotted in triplicate onto the biochip surface) during the enrichment step. SPRi data are treated and plotted as variations of light reflectivity (ΔR (%)) over time for each region-of-interest (corresponding to antibody spots arrayed on the sensor). Differential SPR images (obtained by subtracting a reference SPR image recorded at t(0) to any SPR image acquired later in the experiment) may also be displayed.

*Salmonella* spp. strains are among the most frequent bacterial pathogens causing bacteremia in emerging countries^24, 25^. Besides this, HIV-infected populations, which are particularly important in these countries, have an increased risk of developing salmonellosis^26^. The compactness, easily integrated and low price of biosensors justifies the increasing interest for their use in such circumstances. Among the different types of ligands used to target specific bacteria on a biosensor^27^, antibodies showed remarkable performances in pure culture media^21, 22^. For this reason, the detection of *Salmonella enterica* serovar Enteritidis bacteria is carried out on a microarray functionalized with highly specific anti–*Salmonella* Immunoglobulin G (IgG) along with a series of control IgGs. The simultaneous response analysis of different micro-arrayed IgGs enables the assessment of any positive response by comparison to signals recorded on non-specific control IgGs. The general operation scheme for the direct bacterial SPRi-based detection in blood culture is presented in Fig. 1. In this model experiment, controlled amounts of bacteria are used for blood spiking. The sample is then diluted in commercial blood culture bottles, and an aliquot (100 µL) is loaded on an antibody microarray for real-time SPRi monitoring during enrichment. Variations of light reflectivity followed by SPRi are plotted upon time for each micro-arrayed ligand.

### Workability of the SPRi monitoring in pure culture medium

**Figure 2:**
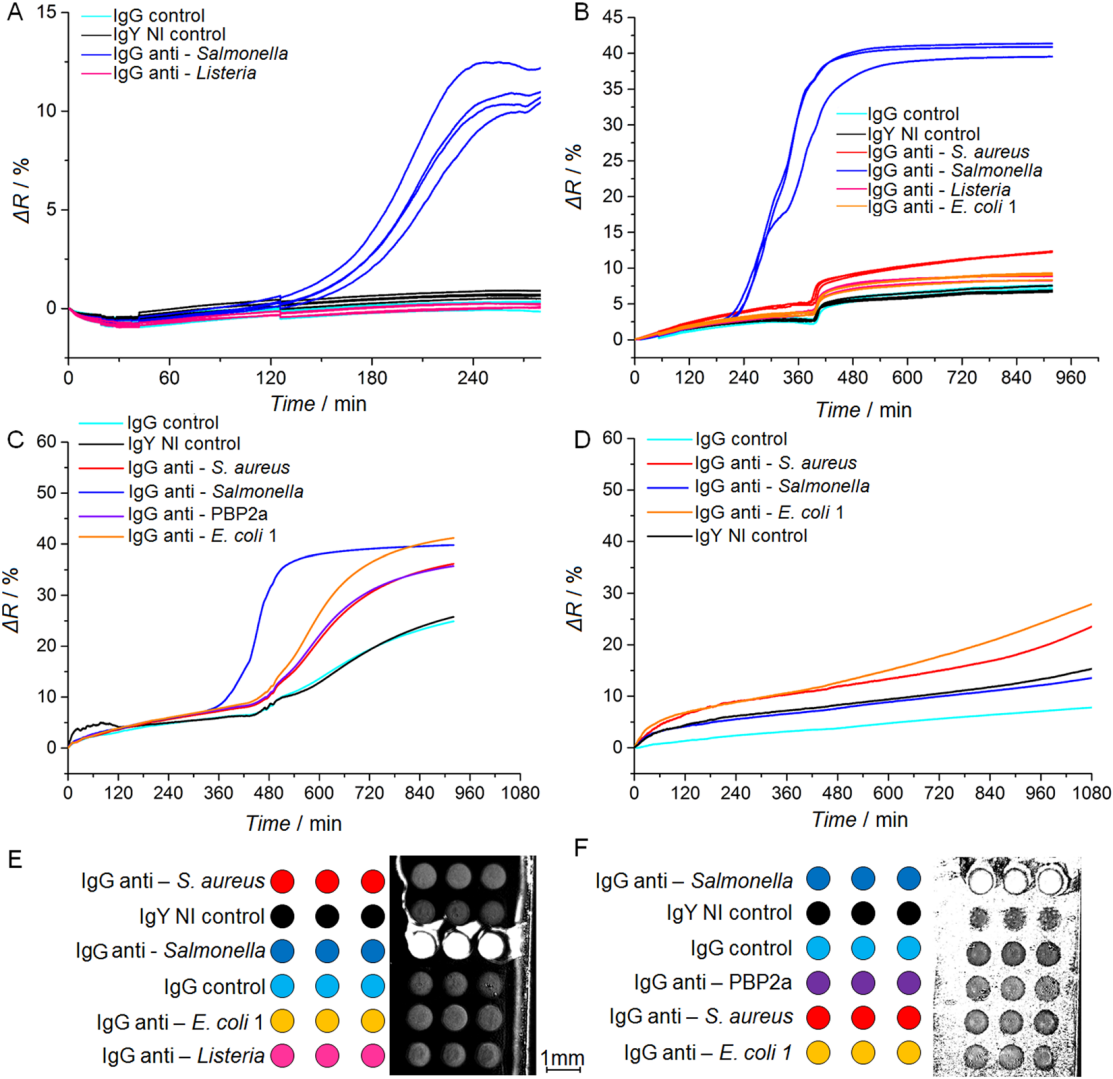
Label-free detection of *Salmonella* Enteritidis contaminating a series of different samples. The SPR imaging of biochip is recorded in pure culture media; in culture media with human serum and in culture media with human blood. (**A**) Pure culture medium spiked with *Salmonella* Enteritidis (9.6 × 10^3^ ± 1 × 10^3^ CFU.mL^−1^ at t = 0). (**B**) *Salmonella* Enteritidis (1.3 × 10^4^ ± 3.3 × 10^3^ CFU.mL^−1^) detection in blood culture media containing human serum (10:1 ratio). (**C**) *Salmonella* Enteritidis (124 ± 16 CFU.mL^−1^) detection in culture media containing human blood (10:1 ratio). (**D**) SPRi data for uncontaminated human blood diluted in culture medium and processed on the antibody microarray. Each curve corresponds to the averaged SPR responses recorded for a triplicate series of each antibody. (**E**) Arraying map and differential SPR image of the biochip incubated in 10% human serum condition (**B**), taken 8 hours after the experiment beginning. (**F**) Arraying map and differential SPR image of the biochip incubated in culture media containing 10% of human blood (**C**) after 8 hours.

Before starting pathogen searches in human blood samples, *S*. Enteritidis detection has been assessed in pure culture medium in absence of blood or other blood component (Fig. 2-A). In such conditions, a significant reflectivity increase was observed 150 min after the incubation started, only on anti-*Salmonella* IgG spots. Other control IgG features did not show any specific response. Raw SPRi data were plotted for each individual spot and confirmed the good reproducibility within each triplicate series arrayed on the same biochip. Untreated SPR data sometimes showed random and unpredictable variations (see the small SPR shift on controls, Fig. 2-B), without apparent correlation with the bacterial concentration, nature of antibodies used or experimental conditions. Such effects might be due to a change in the culture medium optical properties (due to medium content change upon bacterial enrichment) or to the settlement of dead/live bacteria on the biochip. As an intermediate condition before working with whole blood samples, human serum spiked with a controlled amount of *S*. Enteritidis was diluted in commercial media, and incubated in conditions favoring the bacterial growth. SPRi data recorded in real-time did not shown bias due to serum components (Fig. 2-B). After several hours (360 min), only a small positive drift of the SPR signal was observed on negative controls. This late non-specific response could be due to physio-chemical changes of the culture media, induced by bacterial growth. Although such small variations of the refractive index were often observed in SPRi experiments done in diluted serum or blood, they did not prevent the detection of contaminating bacteria. Ultimately, human blood was diluted in commercial medium and spiked with the same bacterial strain before SPR monitoring (Fig. 2-C). Control experiments led in absence of bacteria (sterile condition) have also been completed (Fig. 2-D). These preliminary experiments were performed in 1 mL of human blood diluted in culture media with initial bacterial contaminating levels in the range of 10^2^–10^4^ CFU.mL^−1^. SPR images showing non-specific interactions occurring on the biochip surface exposed to diluted serum or whole blood are presented in Fig. 2-E and F. Obviously, some blood components are interacting with bare gold regions, surrounding the grafted antibodies, which has already been described by others as “Vroman effect”^28^. Interestingly, each antibody spots were spared by such non-specific interactions and thus remained fully functional in presence of blood components. Indeed, unambiguous *S*. Enteritidis SPRi detections on anti-*Salmonella* IgG were completed in blood as can be seen in Fig. 2-F. These preliminary experiments led on small sample volumes (100 µL) validated the SPRi detection of a bacterial pathogen present in blood diluted samples, which is quite remarkable in the light of data published by others on non-specific effects due to blood components and affecting immuno-assays efficiencies^29^. Interestingly, SPRi data monitored for control IgGs in presence (Fig. 2-C) or absence (Fig. 2-D) of bacteria showed different profiles. Indeed, although non-specific to the contaminating bacteria, SPRi responses plotted for the irrelevant IgGs eventually increased after 10 hours, although this response appeared much later than the one observed for specific IgGs. Consequently, a bacterial growth can be detected with this system even if the biochip is lacking antibodies specific to the contaminating bacteria. Our approach thus enables the fast detection of specific bacteria depending on the antibody set arrayed on the biochip, but may also be used to assess the presence of other bacterial contaminant. This property is particularly interesting for qualifying samples where a strict sterility, like blood, is expected.

### Assessment of detection specificity

**Figure 3:**
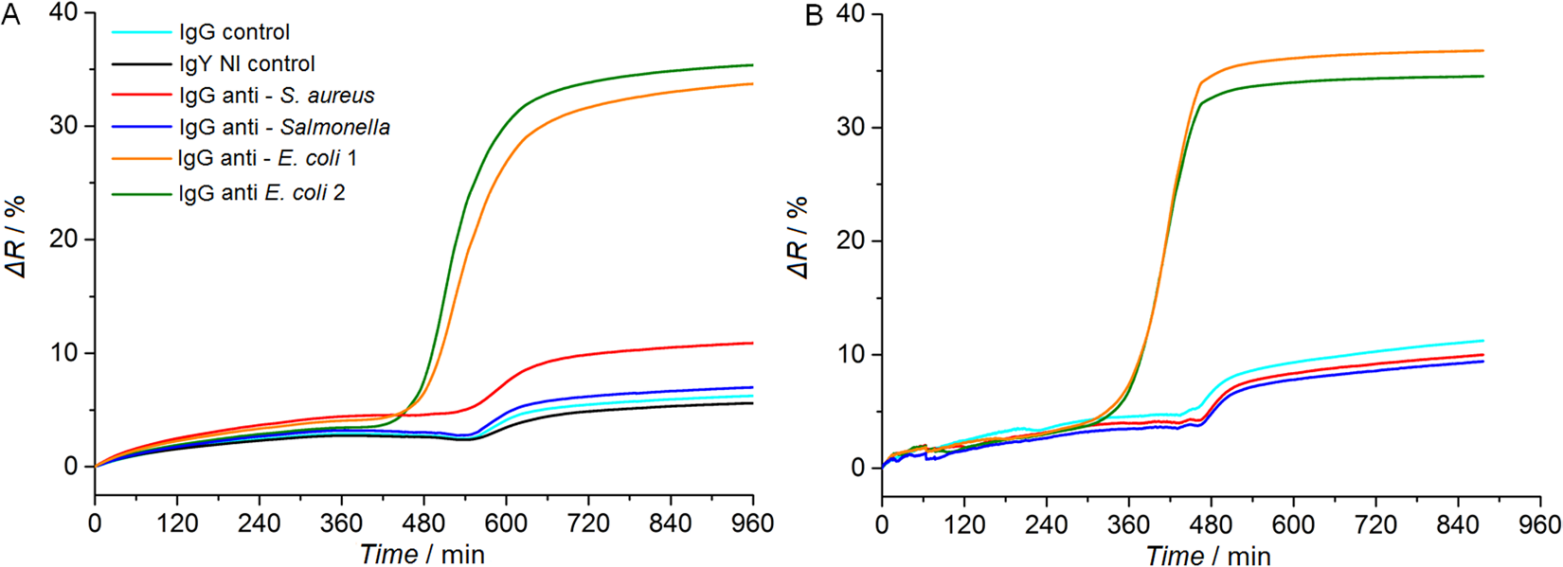
Detection of *E. coli* O157:H7 bacteria contaminating human samples using SPR imaging of multi-strain antibody microarray. An antibody microarray functionalized with two IgGs specific to *E. coli*, as well as IgGs specific to other bacterial strains and control IgGs was prepared and tested in the following conditions: (**A**) Human serum diluted in commercial blood culture medium (1:10 ratio) and spiked with an initial *E. coli* O157:H7 load of 76 ± 11 CFU.mL^−1^; and (**B**) Human blood diluted in commercial blood culture medium (1:10 ratio) and spiked with an initial *E. coli* O157:H7 load of 990 ± 170 CFU.mL^−1^.

In order to assess assay specificity for a targeted bacterial strain, control experiments were completed with *E. coli* O157:H7 bacteria processed on biochip arrayed with anti-*Salmonella*, anti-*S. aureus* and two anti-*E. coli* IgGs. Measured amounts of *E. coli* O157:H7 bacteria were diluted in media containing 10% human serum or human blood, and SPRi monitoring was done during the enrichment step (Fig. 3).

*E. coli* O 157:H7 bacteria were successfully detected by each type of anti-*E. coli* antibodies arrayed on the biochip, in diluted human serum and blood samples. The assay specificity was confirmed as no cross-reactivity between the anti–*Salmonella* IgG spots and anti-*E. coli* O157:H7 bacteria was observed. This approach can then be extended to several pathogenic bacteria detection as long as antibodies specific to each strain are available.

### Growth rate and real-time follow-up of the bacterial multiplication

**Figure 4:**
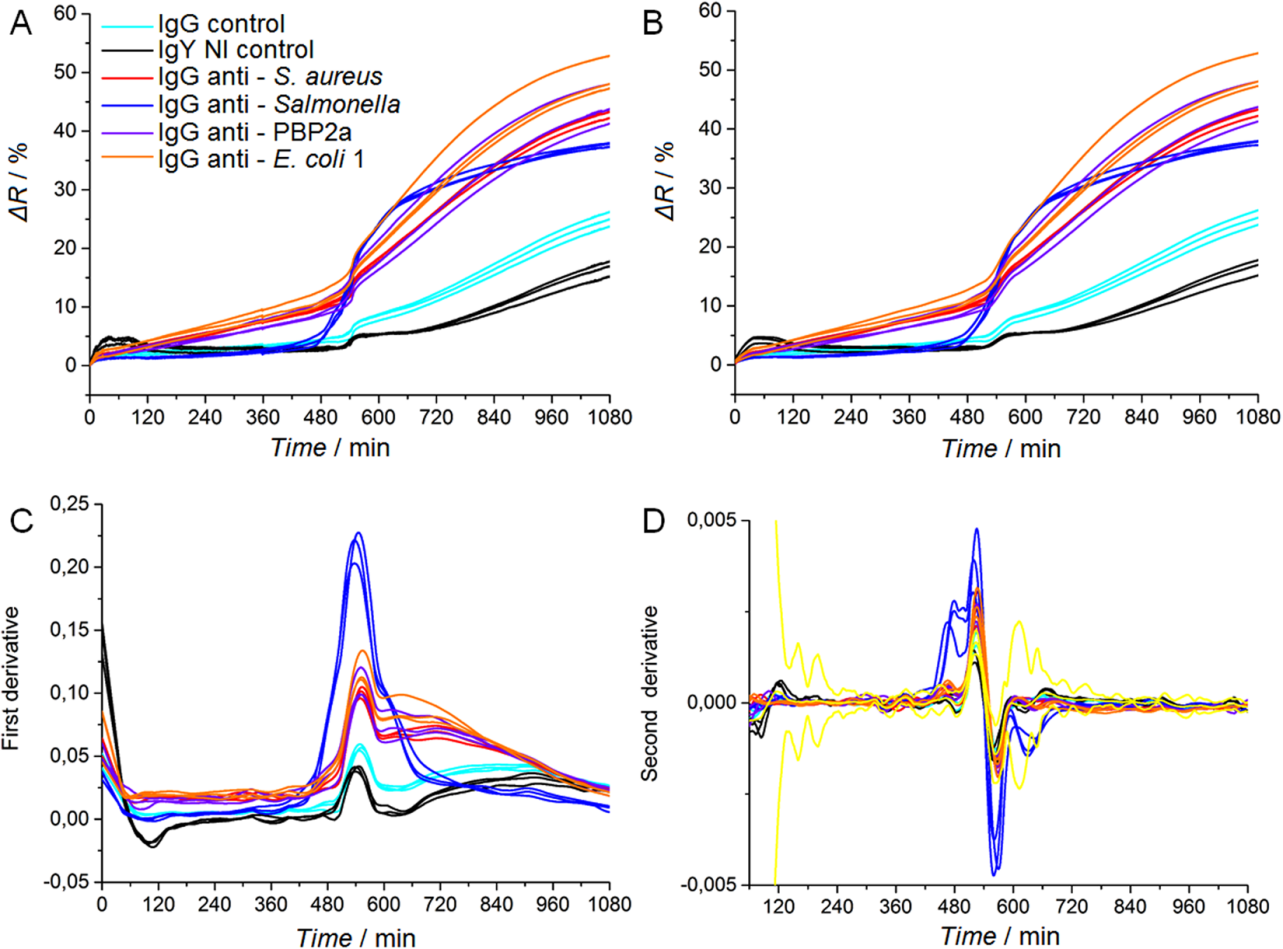
SPRi data analysis and assessment of characteristic detection times. The detection of *Salmonella* Enteritidis, with an initial bacterial load of 14 ± 6 CFU.mL^−1^, is carried in a 1 mL volume composed of blood culture medium and human blood (10:1 ratio). (**A**) Raw SPRi data. (**B**) SPRi data smoothed with a Savitzky-Golay filter. (**C**) First derivatives of SPRi data. (**D**) Second derivatives of SPRi data. Yellow curves represent the averaged SPRi signal of IgY NI and IgG controls plus or minus five standard deviations. These data thus define the threshold values for positivity assessment.

Growth rate values can be calculated from SPRi data measured from similar blood samples contaminated with the same bacteria spiked at different concentrations (respectively N(0_1_) = 124 ± 16 CFU.mL^−1^ and N(0_2_) = 14 ± 6 CFU.mL^−1^ of *S*. Enteritidis, Figs 2-C and 4-A). A 100 min difference is observed in the detection time of *Salmonella* between these two samples contaminated with distinct seeding concentrations. As previously described for real-time monitoring of bacterial growth, characteristic times found for detection can be used to estimate the initial number of viable bacteria present in the sample^22^. Assuming that bacteria are physiologically in exponential growth phase during SPRi monitoring, the growth rate (µ) can be extracted from Equation (1). This equation expresses the number of bacteria in the sample at “t”, depending on the initial contaminating level and growth rate:

1 $${\rm{N}}({\rm{t}})={\rm{N}}(0)\times \exp (\mu \times {\rm{t}})$$

Two assumptions are needed to estimate the growth rate. The first one is that in both samples (same blood sample and same contaminating bacteria) the growth rate is the same. The second one is to assume that the bacteria number N(t) in both samples is identical when the interactions on specific antibodies became distinguishable on each assay, at t_1_ and t_2_ for N(0_1_) = 124 ± 16 CFU.mL^−1^ and N(0_2_) = 14 ± 6 CFU.mL^−1^ respectively. With this two hypothesis, the following equality can be expressed Equation (2):

2 $${\rm{N}}({0}_{1})\times \exp (\mu \times {{\rm{t}}}_{1})={\rm{N}}({0}_{2})\times \exp (\mu \times {{\rm{t}}}_{2})$$

Using the data shown in Figs 2-C and 4-A, we found t_2_ – t_1_ = 100 min. Equation (3), expressing the growth rate, can thus be obtained from Equation (2):

3 $${\rm{\mu }}=\frac{1}{{{\rm{t}}}_{2}-{{\rm{t}}}_{1}\,}\times \,\mathrm{ln}(\frac{{\rm{N}}({0}_{1})}{{\rm{N}}({0}_{2})})$$

In such assay conditions, the calculated growth rate is 0.02 min^−1^, corresponding to a generation time of 33.9 min. This value obtained from biochip analysis is actually higher than other data found in the literature for fully-liquid culture phases (generation time of *Salmonella* growing in exponential phase is around 20.4 ± 0.8 min^−1^)^30^. The difference between both values might be due to several factors such as: growth rate calculation in different culture media;^30^ occurring of a lag phase before exponential growth in the blood culture medium, which may delay the detection time and thus increase the calculated growth rate; growth rate estimation are usually based on optical density measurements of planktonic bacteria, which might be different from growth rates calculated from SPRi signals recorded on the surface of microarrays. Nevertheless, our approach successfully enabled the assessment of consistent bacteria growth rate values in human blood-derived samples.

### SPRi data analysis and positive response assessment

Most SPRi signals recorded from samples contaminated with low levels of bacteria eventually increased for every arrayed antibody, whatever their specificity (Fig. 4-A **)**. In such conditions, a clear distinction between positive and non-specific responses turned out to be way more challenging than with blood-derived samples contaminated with higher pathogen levels. The SPRi signal drift is obviously due to non-specific interactions of blood components with both the gold surface and the micro-arrayed antibodies. Due to the low levels of bacteria initially present in a sample, the SPRi follow-up of bacterial growth may then be shielded by the steady signal drift induced by interactions of blood components with the grafted antibodies, whatever their specificity. To circumvent this problem, a simple data treatment was set to unambiguously distinguish positive SPRi signals from noise or non-specific signals (Fig. 4 and Supplementary Figures S1 and S2). Figure 4-A represents raw SPRi data, while Fig. 4-B corresponds to the smoothed data using a Savitzky-Golay filter prior to derivative calculations. As shown in this figure, such treatment did not significantly modify the curves by comparison to untreated data, and thus does not introduces any bias in the SPR response processing. In presence of low levels of contaminating *Salmonella*, SPRi data first derivative enabled a clear distinction between the anti–*Salmonella* IgG signal and control IgGs (Fig. 2-C), while SPR responses collected from uncontaminated samples did not (Fig. 2-D). Indeed, anti–*Salmonella* IgG signals derivative variations over time were the most important shifts observed among all the arrayed antibodies. The maximum value of the first derivative, corresponding to the inflection point, has thus been used for defining a characteristic detection time^31^. Interestingly, the detection time found with this data treatment was about 520 min, while a clear difference between positive spots and controls was perceptible in raw data as early as 480 min. By plotting the second derivative (Fig. 2-D), the detection time assessment can be improved since the anti–*Salmonella* IgG signal overpasses the threshold at 450 min. This further analysis thus emphasized kinetic differences between positive and negative SPRi signals. Data extracted from Fig. 2-C **(**corresponding to 124 ± 16 CFU.mL^−1^ of *S*. Enteritidis contaminating a human blood sample) were processed the same way (Supplementary Figure S1) and showed similar results. As expected, no conclusive characteristic times could be calculated from uncontaminated samples (Supplementary Figure S2). Indeed, in absence of bacteria, no SPRi signal exceeded the threshold value calculated in the second derivative, thus confirming the sample sterility. In conclusion, this rather simple SPRi data treatment rendered signal positivity more obvious on positive IgGs and also shortened the delay for contamination assessment.

### Bacteria detection in clinical conditions

**Figure 5:**
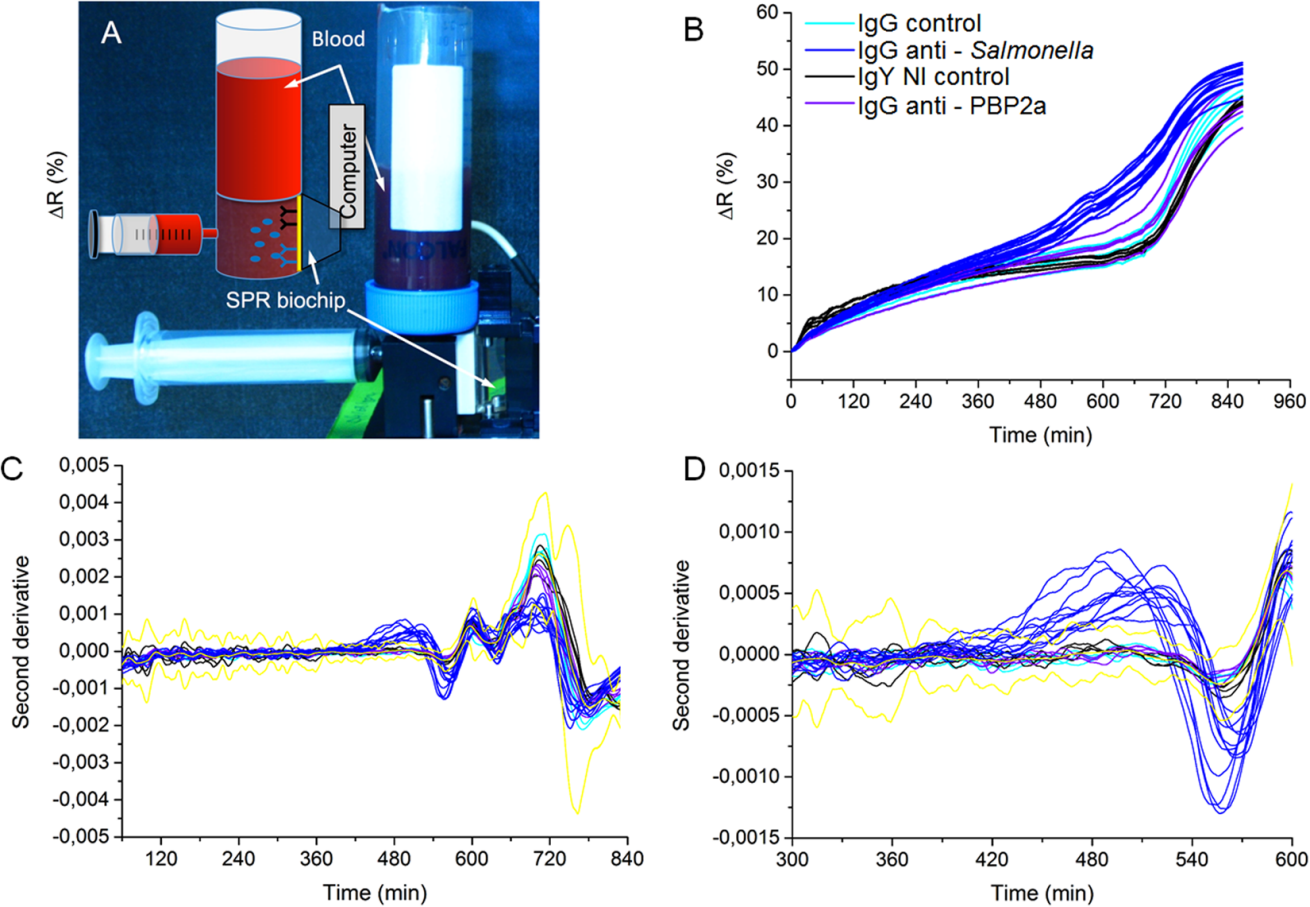
*Salmonella* Enteritidis SPRi detection in large volumes similar to clinical routine analysis (5.4 mL of blood diluted in 26.6 mL of culture medium, for a 1:5 blood-to-broth ratio and a 32 mL total volume). (**A**) Photograph and scheme of the whole set-up containing the sample container, the syringe for reactor filling and the biochip. The instrument and samples were placed in an incubator at 37 °C. (**B–D**) SPRi data recording during the detection of 0.7 ± 0.02 CFU of *Salmonella* Enteritidis per milliliter of blood diluted in blood culture media with a 1:5 ratio (5.4 mL of blood diluted in 26.6 mL of culture medium) in 32 mL total volume. (**B**) Raw SPRi data for pathogen specific antibodies (IgG anti-*Salmonella)* and negative controls (IgG control, IgY NI control and IgG anti-PBP2a). Each IgG has been replicated on the microarray, and every single IgG spot response was independently followed. (**C**) Second SPRi derivatives for every feature arrayed on the biochip. (**D**) Second SPRi derivatives plotted between 300 and 600 minutes. Yellow curves represent the average signal of the control plus or minus five standard deviations.

Next to the successful detection of pathogenic bacteria contaminating human serum and blood in small volumes (1 mL), our approach is tested in experimental sampling conditions similar to the clinical settings. Standard protocols found in clinical settings usually require the collection of 10 to 20 mL of blood, then diluted in liquid culture medium up to a 50 mL final volume. Regarding to the contaminating levels usually found in patients suffering from bacteremia^8, 9^, it is reasonable to expect 1–10 bacteria per blood mL, which means 10–200 bacteria in 50 mL of diluted blood. The blood sample diluted in commercial culture medium was loaded in a 50 mL Falcon tube fitting to a PEEK-drilled chamber (Fig. 5 **)**. This chamber was also connected to a biochip holder-module as well as to a syringe (for sample aspiration and flowing on the microarray). Once assembled, the set up formed a watertight 50 mL large reactor suitable for SPRi detection. Just after sample loading and before the beginning of the SPRi recording, few back-and-forth movements of the syringe piston ensured sample homogenization. The whole set-up was placed in a thermalized incubator to favor bacterial growth. First, the pathogen detection feasibility in “large” sample volume (*i.e*. 6 mL of blood diluted in 24 mL of culture medium containing 43 ± 3 CFU.mL^−1^ of *S*. Enteritidis) has been successfully tested (Supplementary Figure S3). The specific detection of contaminating *Salmonella* was confirmed in only few hours, which validated the suitability of our approach for one-step bacteria detection in “large” samples. To meet the natural contaminating levels found in clinical samples, similar experiments were carried out in large volumes (5 to 6 mL of blood diluted in about 30 mL total volume) but with much lower bacterial contaminating levels. The results are presented in Fig. 5. In this experiment, human blood and blood culture medium were mixed together according to a blood-to-broth ratio of 1:5 in a 32 mL final volume. The initial contaminating bacterial concentration in the total volume was 0.12 ± 0.003 CFU.mL^−1^ corresponding to less than four bacteria in the final volume (5.4 mL of blood diluted in 26.6 mL of culture medium, total volume of 32 mL containing 0.7 ± 0.02 CFU of *Salmonella* Enteritidis) to be processed. *S*. Enteritidis SPRi detection was completed in less than 10 hours and confirmed the successful detection of the pathogen on the antibody microarray. The biochip was arrayed with a series of antibodies spotted in replicates to assess inter-feature variability. As can ben seen on Fig. 5-B, in a biological sample as complex as human blood, the direct assessment of positive contamination by a bacterial pathogen might be challenging on the untreated SPR data (Fig. 5-B). For such sample, plotting of second derivatives is a simple, though efficient, way to assess positivity (Figs 5-C and 5-D). Indeed, this data treatment brings to light positive responses by comparison to the SPR signal from controls, plus or minus five standard deviations. These results confirmed the compatibility of our approach with both the blood culture volumes and pathogen contaminating levels found in clinical conditions.

## Discussion

With the increasing occurrence of multidrug resistant bacterial strains, the early detection of bacteria in clinics is a crucial issue in terms of patient validated prognosis and healthcare cost control. So far, current protocols used in clinics require a two-step process involving an enrichment step, followed by an analysis step. The whole process may last from 24 hours to several days to confirm a bacteremia. In these sequential protocols, the first step aims at increasing the concentration of bacteria present in the sample so that the final number of cells enables an unambiguous detection in the following analysis step. Indeed, usual contaminating levels of bacteria found in patients with bacteremia are so low that no technique has been described so far for one-step detection. SPRi is an optical technique enabling the real-time and label-free monitoring of interactions occurring on a biosensor. This optical technique is also totally independent of the sample optical density as the underlying optical process is based on evanescent wave propagation on a solid surface^32^. This property then makes SPRi an attractive method for monitoring molecular or cellular binding events on a sensor, even though the sample is complex or opaque. For this reason, we designed a SPRi-based method for blood processing and monitoring of bacteria capture on specific IgGs grafted on a biosensor. The possibility to micro-array, on a single biochip, several antibodies specific to different bacterial strains also enables the identification of several pathogens causing bacteremia on a single device^21^. The set-up was designed to enable the real-time monitoring of bacteria multiplication using SPR imaging. The simultaneous coupling of the enrichment step with the SPRi analysis steps significantly shortens the overall process duration and enables the detection of relevant pathogenic bacteria present in highly complex samples such as human serum and blood. Another advantage of this one-step alternative for blood analysis is its ability to detect very low levels of bacteria (only few cells) present in volumes similar to those used in standard blood cultures (more than 30 mL). Such strategy assayed on standardized commercial media spiked with controlled amounts of bacteria enabled the plotting of calibration curves^21^. With such reference data, initial bacterial loads could be extracted. The specific detection of less than one *S*. Enteritidis bacterium per milliliter of blood, in a large volume, confirms the full compatibility of this method with clinical setting requirements. Neither blood components nor blood culture media inhibit bacterial detection (Figs 2 and 3) although, sometimes, some non-specific response eventually appears for the microarrayed IgGs, whatever the antibody specificity (Figs 2-Cand 4-A). This phenomenon usually appeared on samples containing low levels of contaminating bacteria. In this context, a dedicated SPRi signal analysis treatment was set to decipher specific positive signal increases and non-specific responses due to a drift of the medium optical index. This latter shift is obviously due to the solution impoverishment occurring during bacteria growth in un-renewed solution. The plotting of the second derivative of SPRi signals upon time enables the clear distinction between positive detection and non-specific responses. Interestingly, it also means the bacterial growth *per se* induces physio-chemical media modifications detectable by SPRi, independently to the presence of specific antibodies on the array. Such characteristic may then reveal somehow the presence of bacteria in a sample even if pathogen-specific antibodies are lacking on the microarray. Then, even if this approach may fail to identify a bacterial strain in case of lacking specific antibodies arrayed on the biochip, our approach still enables to confirm a bacteremia, which remains critical information for physicians. As it is a non-destructive approach, further analysis -like plating on selective media and antibiotic susceptibility testing- could be conducted right after SPR-based detection, *i.e*. 12 to 24 hours after sampling. On the contrary to SPRi monitoring of bacterial growth in standardized commercial culture medium^21^, the assessment of initial bacterial loads is not relevant in blood samples as the extracted values are highly dependent on the pathogen itself as well as on the natural variability of blood contents. Besides this, on a practical point of view, physicians are requiring faster bacteria detecting methods, rather than initial loads counting tools, as any blood-contaminating bacteria requires antibiotics prescription, whatever the initial contaminating level. Our approach is currently extended to other relevant pathogens in order to develop broad-spectrum microarrays, based on the availability of antibodies targeting bacterial surface antigens. This method will then be simultaneously compared to reference methods to assess the biosensing performances. Eventually, the possibility to perform antibiotic susceptibility testing and identification in the same time is also envisioned, as it would be a major advantage for faster and complete diagnosis. Another potential improvement of our method would be the extension of such approach to other human biological samples (urine, cerebro-spinal fluids, broncho-alveolar fluids, etc.). In such case, we anticipate the need to develop specific culture media enabling the simultaneous bacterial growth and SPRi, to get rid of pathogen culture variabilities in these samples, non-specific side-product adsorption in culture conditions, etc. In the specific case of human blood or serum, our approach took benefit of selected culture media developed for several years for optimal bacterial growth in liquid conditions.

## Methods

### Bacterial strains

The *Salmonella enterica* subspecies *enterica* serovar Enteritidis (*S*. Enteritidis) was provided by the “Institut Français d’Hygiène et d’Analyse” (ISHA, Massy, France). The *Escherichia coli* O157:H7 CIP 105917 was obtained from the Pasteur Institute (Paris, France).

### Human blood sampling

Blood samples were provided by the “Etablissement Français du Sang” (EFS, La Tronche, France). Blood donor signed consents were obtained by the EFS prior to sampling. All methods were carried out in accordance with relevant French guidelines and regulations. Blood sampling was directly performed in blood culture bottles (Bactec Aerobic, Becton Dickinson, Le Pont de Claix, France) according to the manufacturer instructions. Human sera were prepared from whole blood drawn in BD Vacutainer® Serum tubes (ref 369032, Becton Dickinson, Le Pont de Claix, France). After filling, the tubes were kept vertically for 45 min at room temperature to activate clotting. Then, they were centrifuged (10 min, 13 000 g at room temperature with a slow deceleration). Serum, confined in the yellow upper phase, was pipetted under a laminar biosafety cabinet and immediately frozen at −80 °C. Samples were thawed prior to each SPRi experiment.

### Culture conditions

Before each experiment, an isolated colony of bacterial pathogen cultivated on Tryptone Soy Agar plate (TSA, bioMérieux, Lyon, France) was resuspended in 4 mL of sterile Tryptic Soy Broth (TSB, Sigma, Saint Quentin Fallavier, France). Suspensions were then incubated overnight at 37 °C under constant agitation (150 rpm). Then, ten-fold serial dilutions of the initial bacterial suspension were prepared in blood culture media. 100 µL of the 10^−6^ and 10^−7^ dilutions were plated on TSA (in triplicate) to determine the exact initial bacterial count. For each experiment, 100 µL of the ten-fold dilution were used for sample spiking. Initial bacterial concentrations were determined the next day by manual colony counting. For each assay, control experiments were done without bacteria.

### Antibody microarraying and SPRi monitoring

A mouse monoclonal antibody, hereafter referred as anti–*Salmonella* IgG (provided by H. Volland, CEA Saclay, France), was used for specific *S*. Enteritidis detection^21^. All other antibodies listed below were used as negative controls for *S*. Enteritidis detection experiments. KLH, a mouse monoclonal antibody, referred as “IgG negative control” was given by L. Bellanger (CEA Marcoule, France). Antibodies Ab11439, Ab17473, Ab31499 were purchased from Abcam (Cambridge, United-Kingdom) and respectively named “IgG anti – *Listeria*”, “IgY NI control” and “IgG anti – *E. coli* 1”. The “IgY NI control” is a chicken IgY antibody recognizing the human protein beta-amyloid. The second antibody recognizing *E. coli* (AbD Serotec 4329–4306) was purchased from BioRad (Kidlington, United-Kingdom) and referred as “IgG anti – *E. coli* 2”. Finally, two other IgGs were also used: KPL (ref 01–90–05 Kirkegaard & Perry Laboratories, Gaithersburg, United-States) and 10-P08A (Interchim, Montluçon, France). They were respectively named “IgG anti – *S. aureus*” and “IgG anti – PBP2a”. All antibodies were covalently coupled to pyrrole monomers in a reaction involving primary amine functions of the protein according to a simple protocol described elsewhere^33^. IgGs were then electro-polymerized on gold-covered SPRi biochips (Horiba Scientific, Palaiseau, France). Briefly, antibody-pyrrole conjugates (1 µM) were mixed with 20 mM free pyrrole and arrayed, at least in triplicate, on the SPRi biochip using an arraying robot^21^. Each spot diameter was about 650 µm in diameter. Biochips were then used immediately after arraying or stored at 4 °C in PBS (Sigma-Aldrich, France) for few days. A sterile solution of PBS + 1% BSA (Bovine Serum Albumin, A7906, Sigma-Aldrich, France) was systematically used as a blocking solution and incubated on the gold surface 20 min before the beginning of the experiment. The surface was then rinsed with 3 mL of sterile PBS and 1 mL of sterile culture medium before biochip loading in the SPRi apparatus. Interactions of the bacteria captured on the biochip were observed in real-time with a SPRi-Lab + system (Horiba Scientific, Palaiseau, France).

### Data treatment

Raw SPRi data differences were usually sufficient to clearly distinguish positive signals from non-specific signals on control antibodies as maximum reflectivity shifts are dependent on several parameters such as the sample intrinsic optical properties and bacterial concentrations but also on gold layer thickness (covering the biochip) or on the polarized exciting wavelength. In blood containing medium, noise levels were usually higher and more fluctuant than in pure liquid broth and thus required data treatment. After data smoothing using a Savitzky–Golay filter with a 1500 points period, first and second derivatives of the SPR signals were plotted upon time using the Origin Software (Origin Software, San Clemente, CA, USA). In case of an exponential curve characteristic to the bacterial growth, the second derivative is positive. A positivity threshold was set such that an antibody response was considered as positive if the highest value of the second derivative exceeds the control averaged level (KLH and IgY non immune), plus or minus five standard deviations.

## Electronic supplementary material

Supplementary file
