# Supplementary material for: Biochips for Direct Detection and Identification of Bacteria in Blood Culture-Like Conditions
Source: Sci Rep. 2017 Aug 25;7:9457. doi: 10.1038/s41598-017-10072-z (PMC5572712; doi:10.1038/s41598-017-10072-z)
Supplement: Supplementary file 1 — Supplementary file [file 41598_2017_10072_MOESM1_ESM.pdf]

Supplementary information

Biochips for Direct Detection and Identification of Bacteria in Blood Culture-Like Conditions

V. Templier[1], T. Livache[1], S. Boisset[2,3], M. Maurin[2,3], S. Slimani[1], R. Mathey[1], Y. Roupioz[1*]

[1] Univ. Grenoble Alpes, CNRS, CEA, INAC, SYMMES, F-38000 Grenoble

[2] Laboratoire de Bactériologie, Centre Hospitalier Universitaire Grenoble Alpes, Univ. Grenoble Alpes, F-38000 Grenoble, France.

[3] Equipe Therex, TIMC-IMAG, CNRS UMR 5525, Univ. Grenoble Alpes, F-38000 Grenoble, France.

Corresponding author: Y. Roupioz, yoann.roupioz@cea.fr

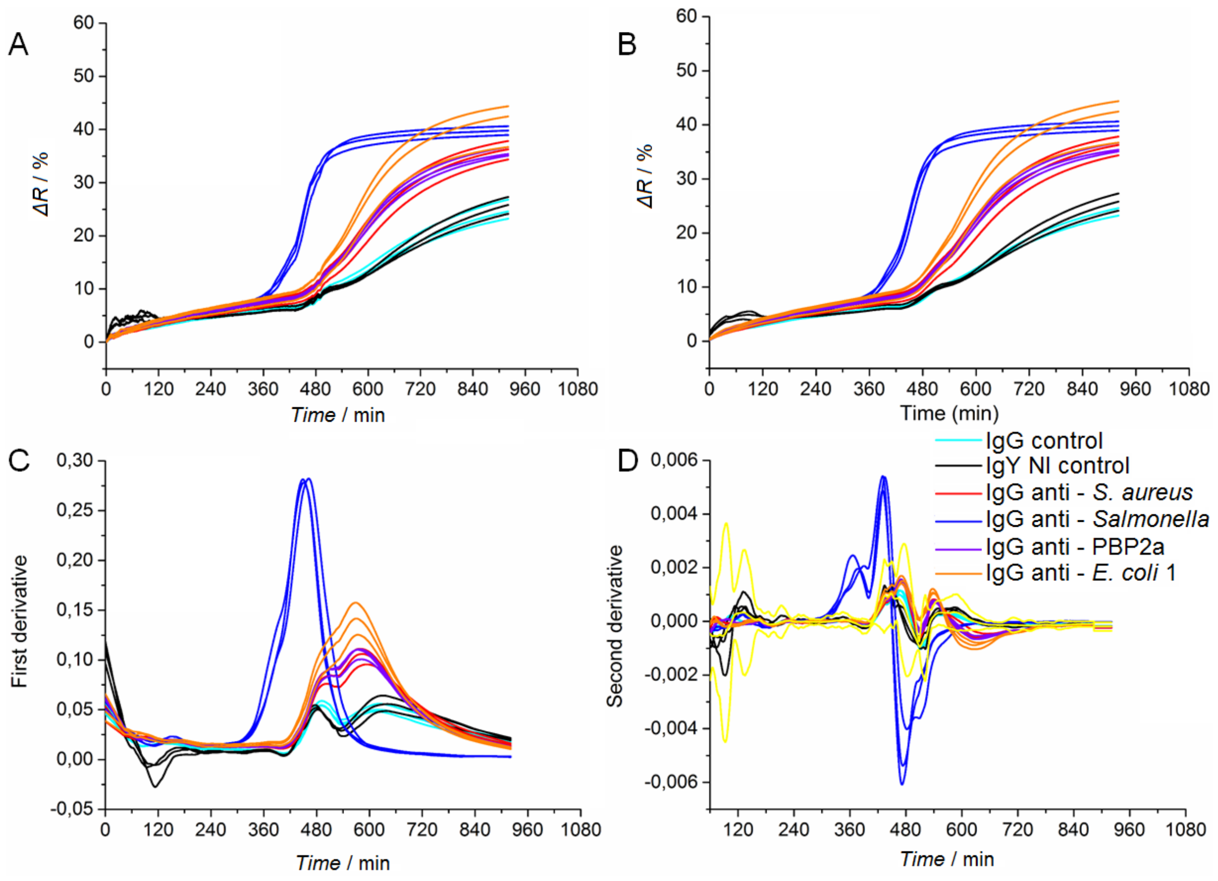

Figure S1: SPRi data analysis for an initial bacterial load of 124 ± 16 CFU.mL^{-1} of *Salmonella* Enteritidis in 1 mL culture media containing human blood (10:1 ratio). **A)** Raw SPRi data. **B)** Smoothed SPRi data. **C)** First derivatives calculated from SPRi data. **D)** Second derivatives caculated from SPRi data. Yellow curves represent the averaged signal of the control plus or minus five standard deviations.

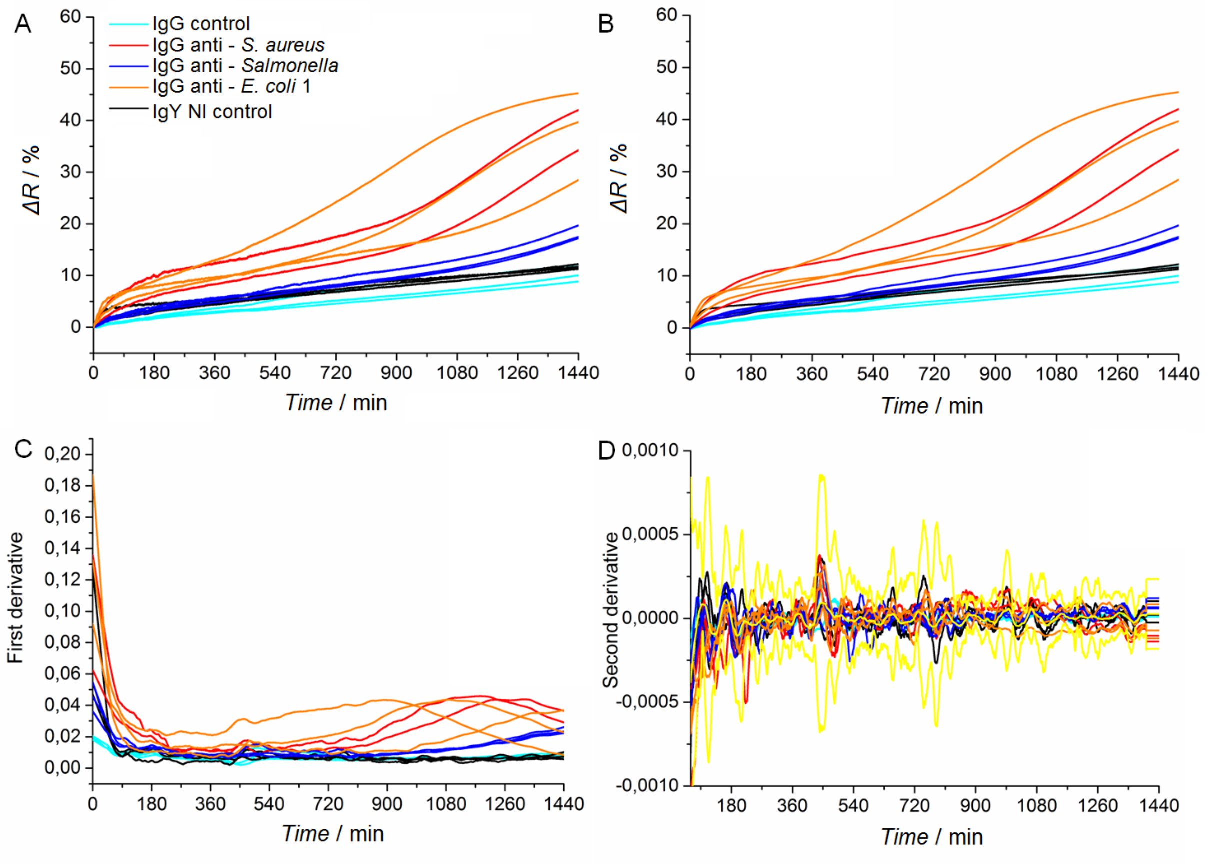

Figure S2: SPRi data treatment for the sterile control in experimental conditions corresponding to the Figure 2-D (culture medium and human blood in a 10:1 ratio). **A)** Raw SPRi data. **B)** Smoothed SPRi data. **C)** First SPRi derivative. **D)** Second SPRi derivative. Yellow curves represent the averaged SPR signal of the control plus or minus five standard deviations.

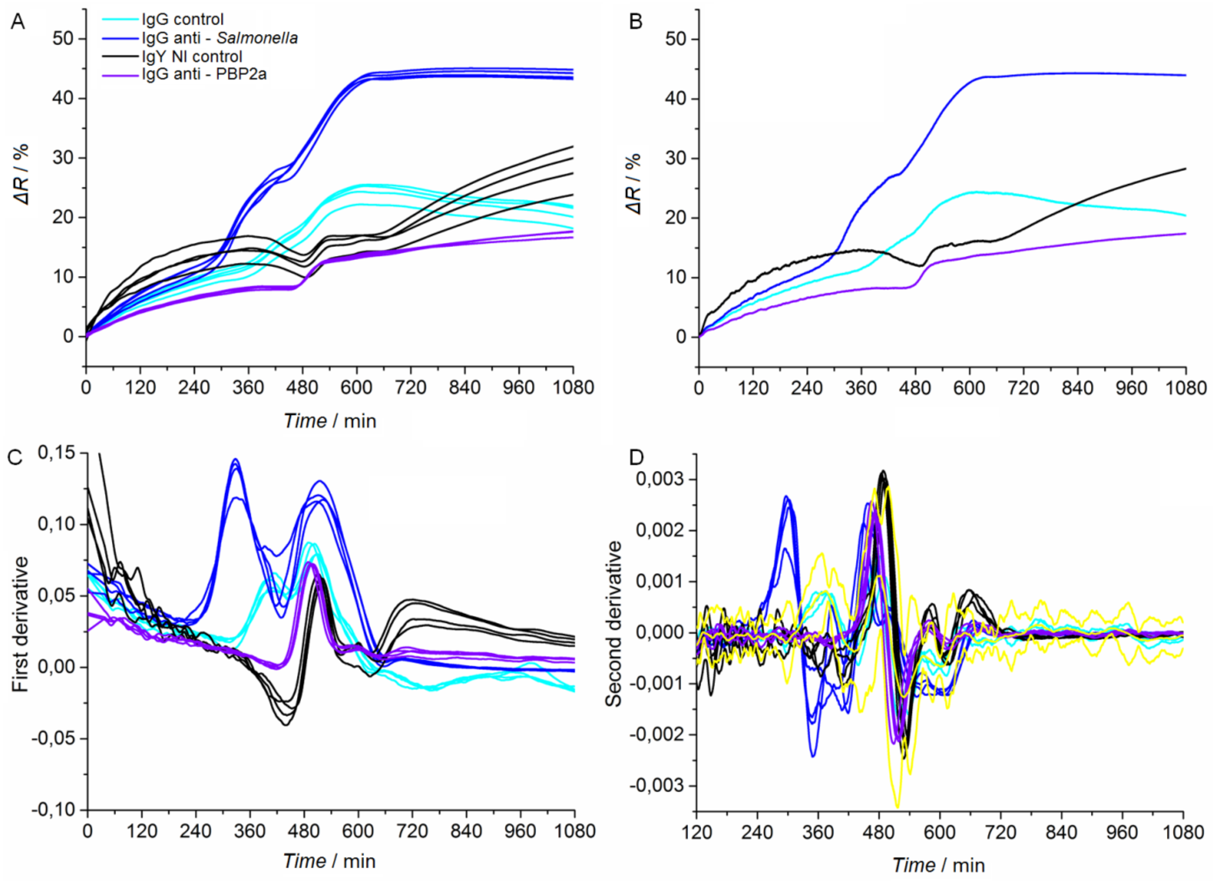

Figure S3: SPRi data treatment for the detection of *Salmonella* Enteritidis in blood culture-like conditions (*i.e.* 6 mL of blood diluted in 24 mL of culture medium, giving a 1:5 blood-to-broth ratio and a final volume of 30 mL). The initial bacterial load is 43 ± 3 CFU.mL^{-1} representing approximately 260 bacteria per blood milliliter. **A)** Smoothed SPRi signals. **B)** Averaged SPRi data for the triplicates. **C)** First SPRi derivatives. **D)** Second SPRi derivatives. Yellow curves represent the averaged SPRi signal of the control plus or minus five standard deviations.
